# Controlling Dynamic DNA Reactions at the Surface of Single-Walled Carbon Nanotube Electrodes to Design Hybridization Platforms with a Specific Amperometric Readout

**DOI:** 10.1021/acs.analchem.1c05294

**Published:** 2022-03-18

**Authors:** Simone Fortunati, Ilaria Vasini, Marco Giannetto, Monica Mattarozzi, Alessandro Porchetta, Alessandro Bertucci, Maria Careri

**Affiliations:** † Department of Chemistry, Life Sciences, and Environmental Sustainability, University of Parma, 43124 Parma, Italy; ‡ Department of Chemical Sciences, University of Rome Tor Vergata, 00133 Rome, Italy

## Abstract

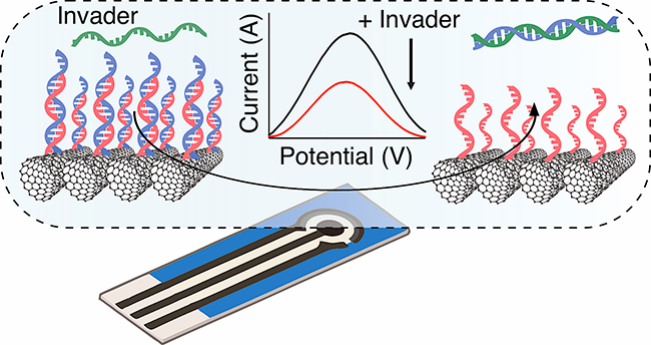

Carbon nanotube (CNT)-based electrodes are cheap, highly performing, and robust platforms for the fabrication of electrochemical sensors. Engineering programmable DNA nanotechnologies on the CNT surface can support the construction of new electrochemical DNA sensors providing an amperometric output in response to biomolecular recognition. This is a significant challenge, since it requires gaining control of specific hybridization processes and functional DNA systems at the interface, while limiting DNA physisorption on the electrode surface, which contributes to nonspecific signal. In this study, we provide design rules to program dynamic DNA structures at the surface of single-walled carbon nanotubes electrodes, showing that specific DNA interactions can be monitored through measurement of the current signal provided by redox-tagged DNA strands. We propose the use of pyrene as a backfilling agent to reduce nonspecific adsorption of reporter DNA strands and demonstrate the controlled formation of DNA duplexes on the electrode surface, which we then apply in the design and conduction of programmable DNA strand displacement reactions. Expanding on this aspect, we report the development of novel amperometric hybridization platforms based on artificial DNA structures templated by the small molecule melamine. These platforms enable dynamic strand exchange reactions orthogonal to conventional toehold-mediated strand displacement and may support new strategies in electrochemical sensing of biomolecular targets, combining the physicochemical properties of nanostructured carbon-based materials with programmable nucleic acid hybridization.

## Introduction

Electrochemical sensors that use specific sequences of synthetic DNA as molecular receptors and DNA-based systems for signal generation and amplification enable the rapid detection of target molecules by converting programmable hybridization and specific biomolecular recognition into measurable electrical outputs.^1,2^ Electrochemical DNA sensors harnessing redox-tagged DNA probes (E-DNA) anchored to gold electrodes have been engineered into point-of-care diagnostic devices enabling rapid, reagentless, amperometric detection of clinically relevant small molecules, nucleic acids, and proteins.^3−6^ Strategies borrowed from DNA nanotechnology based on dynamic DNA structures and programmable reactions have bolstered a wide diversity in DNA recognition interfaces with improved sensitivity and specificity.^7−16^ A precise control of the physicochemical properties and of the biomolecular recognition processes at the DNA–electrode interface is key to robust and efficient E-DNA platforms. This includes an optimization of the immobilization protocols, a control of the probe density and orientation, a minimization of unwanted nonspecific adsorption on the electrode surface, and a rational design of the molecular architecture.^17−20^ Carbon nanotube screen-printed electrodes (CNT-SPEs) are a promising electrochemical platform because of their reduced cost compared with more widely used gold electrodes, their enhanced amplification of electrochemical signals based on their high conductivity, their large surface area that can accommodate an increased number of bioreceptors and their intrinsic electrocatalytic properties promoting electron transfer processes at the interface.^21−24^ CNT surfaces give stable π–π interactions with the nucleobases of nucleic acids, which has been harnessed to produce biosensors that leverage noncovalent adsorption and target-induced desorption to generate electrochemical outputs.^25−28^ However, sensing strategies built on this approach suffer from difficulties in achieving homogeneous and reproducible interfaces based on nonspecific π–π interactions and are limited to adsorption/desorption processes that do not allow engineering of more complex and dynamic molecular mechanisms. Alternatively, covalent surface functionalization with DNA probes enabled the design of electrochemical hybridization platforms in which molecular processes at the interface were monitored through label-free impedance measurements or voltammetric strategies based on the electrooxidation of nucleobases or intercalators.^29−34^ Current formats of these sensors, though, measure changes in electrochemical properties at the interface rather than transduce sequence-specific molecular recognition events, and therefore, they do not allow monitoring of dynamic DNA reactions such as strand exchange processes. The strong noncovalent interactions between nucleic acids and carbon nanotubes also pose a substantial challenge in the design of E-DNAs based on CNT-SPEs, which requires an analysis of the extent to which nonspecific signal can extra-contribute to the expected target-induced amperometric output. Recently, Fortunati et al. and Liu et al. showed that dynamic nucleic acid systems and higher-order hybridization reactions can be interfaced with CNT-SPEs to develop amperometric biosensors in which the resulting current signal is ultimately generated through multistep, reagent-intensive, enzymatic processes.^35,36^ To the best of our knowledge, CNT-SPE-based E-DNAs that allow direct amperometric monitoring of specific nucleic acid hybridization events have not been reported yet. In this context, we provide here design rules to program and control dynamic DNA structures at the surface of single-walled carbon nanotube screen-printed electrodes (SWCNT-SPEs) that enable direct amperometric monitoring of hybridization processes. We show how to reduce unwanted nonspecific interactions, demonstrate conduction and detection of programmable DNA strand displacement reactions, and then extend our approach to the formation and manipulation of recently discovered noncanonical DNA structures that can be used in the design of novel amperometric hybridization platforms.

## Materials and Methods

Trizma base, sodium hydroxide (NaOH), hydrochloric acid (37% w/v; HCl), acetic acid, dimethyl sulfoxide (DMSO), magnesium acetate tetrahydrate, magnesium chloride (MgCl_2_), *N*-(3-dimethyl)-*N*′-ethylcarbodiimide hydrochloride (EDC), *N*-hydroxysuccinimide (NHS), sodium bicarbonate (NaHCO_3_), sodium dodecyl sulfate (SDS), ethylendiaminetetraacetic acid disodium salt dihydrate (EDTA), 4-morpholineethanesulfonic acid monohydrate (MES), pyrene, melamine, streptavidin–alkaline phosphatase from *Streptomyces avidinii* (ALP-Strp), Tween 20, and bovine serum albumin (BSA) were purchased from Merck (Milan, Italy). Hydroquinone diphosphate (HQDP) and single-walled, carbon nanotube screen-printed electrodes (DropSens DRP-110SWCNT) were purchased from Metrohm Italiana s.r.l. (Origgio, Italy). Synthetic DNA oligonucleotides, including biotinylated, amine-modified, Atto-MB2-modified, and Cy3-modified oligonucleotides, were purchased from Metabion (Germany).

### Electrochemical Measurements

Voltammograms were acquired using a Metrohm Autolab PGSTAT204 equipped with NOVA 2.1.4 software and DRP-DSC boxed connector for SPE, purchased from Metrohm Italiana s.r.l. (Origgio, Italy). Amperometric signal of Atto-MB-2, which derives from the dye methylene blue, was measured by applying a preconditioning potential of −0.6 V for 30 s, followed by differential pulse voltammetry (DPV) analysis performed from −0.6 to 0 V. Hydroquinone amperometric signal was acquired applying a preconditioning potential of −0.5 V for 30 s, followed by DPV analysis performed from −0.5 V to +0.3 V. DPV acquisition parameters for analysis of both redox species were set as follows: step potential = +0.005 V; modulation amplitude = +0.05 V; modulation time = 0.1 s; interval time = 0.4 s. The DPV voltammograms were elaborated using the baseline correction function of NOVA 2.1.4, applying a polynomial algorithm for the determination of current peaks in the nanoampere range.

### Data Treatment

The linear range, limit of detection (LOD), and limit of quantitation (LOQ) were calculated according to “Eurachem Guidelines”,^37^ performing at least three replicated measurements for each concentration level.

### Buffers

TAE buffer: 40 mM Trizma Base, 20 mM acetic acid, 2 mM EDTA, 12.5 mM magnesium acetate; pH was adjusted to 8.0 using 1 M HCl. TRIS buffer saline (TBS): 0.1 M Trizma base, 0.02 M MgCl_2_; pH was adjusted to 7.4 using 1 M HCl. TRIS buffer saline–Tween (TBS-T): 0.1 M Trizma Base, 0.02 M MgCl2, 0.05% w/v Tween 20; pH was adjusted to 7.4 using 1 M HCl. Carbonate buffer (CB): 0.1 M NaHCO_3_, 0.1% w/v SDS; pH was adjusted to 9.0 using 1 M NaOH. Reading buffer (RB): 0.1 M Trizma base, 0.02 M MgCl_2_; pH was adjusted to 9.8 using 0.1 M HCl. MES buffer: 0.1 M MES; pH was adjusted to 5.0 using 1 M NaOH.

### Electrode Functionalization

SWCNT-SPEs were reacted with 50 μL of a 0.2 M EDC and 0.05 M NHS solution in MES buffer. After 30 min the electrodes were thoroughly rinsed with distilled water. Covalent immobilization of capture oligos was carried out incubating the NHS-functionalized electrodes with 50 μL of a 500 nM solution of amine-modified DNA probes in CB for 2 h, followed by washing with distilled water. Backfilling with pyrene was obtained by depositing on the SPE surface 50 μL of a 500 nM solution of pyrene in DMSO for 1 h, followed by washing with DMSO and distilled water.

### Analysis of Surface Functionalization

Functionalization of SWCNT-SPEs was performed using a biotinylated DNA probe. Enzymatic labeling was then carried out through incubation for 15 min with a 10 ng/mL solution of ALP-Strp conjugate containing 20 mg/mL BSA in TBS buffer, followed by washing with TBS-T (1×) and TBS (1×). The amperometric readout was performed by depositing 50 μL of a 1 mg/mL solution of HQDP in reading buffer on the electrode surface for 150 s prior to DPV analysis. Probe density was estimated by performing functionalization of the electrode surface using Cy3-labeled, amine-modified DNA oligonucleotides and measuring the intensity of fluorescence emission of the Cy3 label in solution (λ_ex_ = 540 nm, λ_em_ = 565 nm) on the FluoroMax-3 spectrofluorimeter (HORIBA JobinYvon, Bensheim, Germany).

### DNA Hybridization Studies

Solutions of different concentrations (30, 50, 70, 100, 300, and 500 nM) of Atto-MB2-modified poly(A) DNA strands were prepared in TAE buffer, of which 50 μL were deposited on SWCNT-SPEs previously functionalized with amine-modified poly(T) probes and left incubating for 2 h. Next, the electrode surface was washed with TAE buffer and amperometric measurements were carried out in reading buffer. Control experiments for the evaluation of nonspecific amperometric signal due to DNA physisorption were conducted using a 100 nM solution of noncomplementary Atto-MB2-modified poly(T) strands.

### Toehold-Mediated Strand Displacement Reactions

SWCNT-SPEs functionalized with 15-nt poly(T) DNA probes were reacted with a 100 nM solution of a complementary Atto-MB2-modified poly(A) sequence flanked by a 6-nt-long toehold domain (the full sequences are reported in Section 1 in the Supporting Information) in TAE buffer for 2 h, after which washing with TAE buffer was carried out. Next, strand displacement reactions were performed by incubating the electrode surface for 1 h with 50 μL of different concentrations (50 and 100 nM) of a sequence full complementary to the toehold-bearing strand of the surface duplexes. After washing with TAE buffer, amperometric measurements were conducted in reading buffer. Sequence-specificity experiments were conducted using solutions of invader probes (100 nM) bearing either one or three noncomplementary nucleobases in the toehold region, as well as a completely random sequence. Control experiments for the evaluation of nonspecific amperometric signal due to DNA physisorption were conducted by incubating SWCNT-SPEs functionalized with 15-nt poly(T) DNA probes with corresponding concentrations (50 and 100 nM) of a noncomplementary Atto-MB2-modified poly(T) strand.

### Formation of Poly(T)-Melamine Duplexes on the Electrode Surface

SWCNT-SPEs functionalized with poly(T) DNA probes conjugated with 0, 3, or 6 GC bases were first incubated for 1 h with 25 μL of 200 nM solutions in TAE buffer of the complementary Atto-MB2-modified 15-nt poly(T) sequence (0GC), 15-nt poly(T) sequence conjugated with 3 G/C bases (3GC), or 15-nt poly(T) sequence conjugated with 6 G/C bases (6GC), respectively (see Figure 5b). Next, 25 μL of a 2 mM solution of melamine in TAE were added to a final volume of 50 μL of solution (1 mM melamine, 100 nM of 0GC, 3GC, or 6GC DNA strand, respectively) on the electrode surface, which was left incubating for another 1 h. Washing with TAE buffer was then carried out and amperometric measurements were conducted in reading buffer. In parallel, analogous experiments were carried out without addition of melamine.

### Strand Exchange Reactions Based on Poly(T)-melamine Duplexes

SWCNT-SPEs functionalized with poly(T)-melamine duplexes, assembled using 15(T)-3GC-duplex-forming strands, were incubated for 1 h with 50 μL of a 100 nM solution of a 15-nt poly(A) sequence in TAE buffer. After washing with TAE buffer, amperometric measurements were conducted in reading buffer. Control experiments were conducted by repeating the same experimental steps without addition of melamine.

## Results and Discussion

### Functionalization of SWCNT-SPEs with DNA Probes

**Figure 1:**
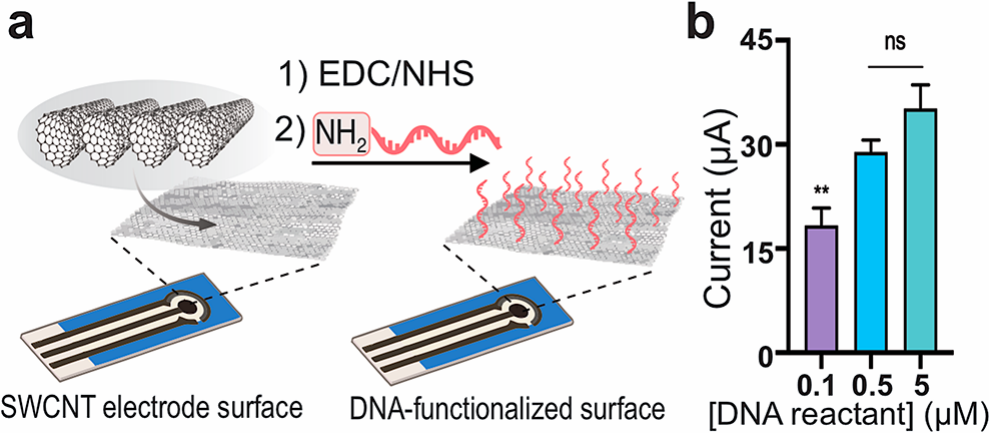
(a) Schematic illustration of the functionalization of SWCNT-SPEs with amine-modified DNA probes. (b) Current signals obtained using different concentrations of biotinylated DNA probe reactant (mean value ± sd, *n* = 3, **p < 0.01, ns = nonsignificant) following incubation with alkaline phosphatase–streptavidin conjugates and its enzymatic substrate.

Functionalization of SWCNT-SPEs with DNA probes through formation of covalent bonds can be obtained through a coupling reaction between the carboxylic acid groups present on the CNT surface and amine-modified oligonucleotides. Electrode surface treatment with EDC and NHS allows for the conversion of carboxylic groups into reactive NHS esters which can efficiently react with a terminal amine group on DNA probes to form stable amide bonds (Figure 1a). We used different concentrations of a 15-mer DNA oligonucleotide (capture probe) conjugated at the 5′-end with an amine group and at 3′-end with biotin (full sequences are reported in Section 1 in the Supporting Information) to modify SWCNT-SPEs and determine the concentration of DNA reactant in order to maximize surface functionalization.

The presence of a biotin molecule at the free 3′-end of the attached oligonucleotides allows for creating a complex with a streptavidin-alkaline phosphatase conjugate, thus, generating an amplified current signal by incubation with HQDP that is converted by the enzyme into electroactive hydroquinone. This is proportional to the amount of DNA probes attached to the electrode surface (Figure S1). As shown in Figure 1b, when a 500 nM concentration of amine-modified DNA oligonucleotides was used for the covalent modification of the electrodes, the resulting current signal was not statistically different (*p* > 0.05) from that obtained using a 5 μM concentration of the same oligos, indicating that a 500 nM concentration of amine-modified DNA reactant is sufficient to maximize the functionalization of SWCNT-SPEs with the desired DNA probes. Probe density expressed as the amount of DNA molecules attached per mm^2^ of electrode surface area was quantified by fluorescence spectroscopy and resulted to be approximately 4.3 × 10^11^ DNA probes/mm^2^, which is higher than the average values reported in the literature for DNA–gold electrode interfaces (Figure S2).^8,38^

### Control of Nonspecific DNA Physisorption on the Electrode Surface

**Figure 2:**
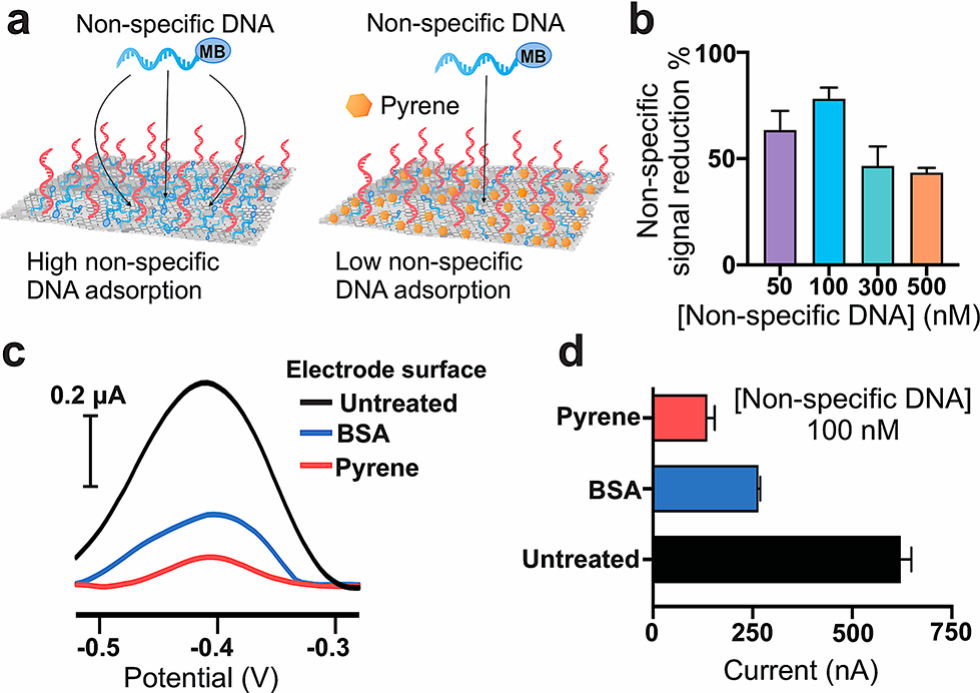
(a) Schematic illustration of the electrode surface treatment with pyrene as a backfilling agent to reduce nonspecific adsorption of DNA strands. MB is the Atto-MB2 redox tag. (b) Nonspecific signal reduction % relative to untreated electrodes when pyrene is used as a backfilling agent at different concentrations of a sample nonspecific DNA strand (mean value ± sd, *n* = 3). (c) Representative voltammograms obtained when redox-tagged nonspecific DNA (100 nM) was incubated on untreated (black curve), BSA-treated (blue curve) and pyrene-treated DNA-functionalized SWCNT-SPEs (red curve), and (d) the corresponding measured current signals (mean value ± sd, *n* = 3).

We then set out to determine the best experimental conditions for the control of specific hybridization processes at the interface. For this purpose, we investigated how to minimize nonspecific physisorption of DNA strands when a sample is left incubating on the CNT electrode, which is a dominant effect when single-stranded DNA is allowed to interact with carbon-based structures.^39^ Our research group recently reported the use of pyrene as a backfilling agent to create a highly hydrophobic layer that is capable of greatly reducing the uncontrolled adsorption of peptide nucleic acid (PNA)-based probes, measured via an enzyme-based amplification process releasing an electroactive species in the working solution.^35^ In the present work, we decided to study the efficiency of this backfilling strategy in the reduction of nonspecific physisorption of DNA-based probes labeled with a redox tag, which supports direct amperometric monitoring of hybridization events. Specifically, we used a model 15-mer DNA oligonucleotide not complementary to the surface DNA probes and conjugated with the Atto-MB2 redox tag to estimate how much of the amperometric signal attributable to nonspecific adsorption of the sample DNA at different concentrations can be reduced by a treatment of pristine SWCNT-SPEs with a pyrene solution in DMSO compared to the signal obtained without backfilling strategies (Figure 2a).

As shown in (Figures 2b, S3 and S4), the use of pyrene as a backfilling agent determined a significant reduction of the amperometric output over the whole DNA concentration range investigated (50–500 nM), enabling a maximum reduction of 78% of the nonspecific current signal with respect to untreated SWCNT-SPEs for a 100 nM DNA sample. Incubation of the electrode surface with BSA, which is conventionally used as a blocking agent for passivation of gold electrodes and surfaces, also enabled significant reduction of the amperometric signal from nonspecific DNA adsorption (Figure 2c,d). However, BSA proved less effective than pyrene in reducing the nonspecific signal caused by DNA physisorption (57% vs 78% reduction of current signal with respect to pristine electrodes), which would lead to a lower sensitivity of the sensing platform. These results demonstrates that the nonspecific signal due to uncontrolled DNA physisorption cannot be eliminated and must therefore be considered when measuring and analyzing the amperometric outputs of E-DNAs based on SWCNT-SPEs. Treatment of the electrode surface with pyrene enabled a significant and reproducible reduction of this effect, so we included it as a standard operation in the preparation of DNA-functionalized SWCNT-SPEs.

### Formation of Specific DNA Duplexes on the CNT Surface

**Figure 3:**
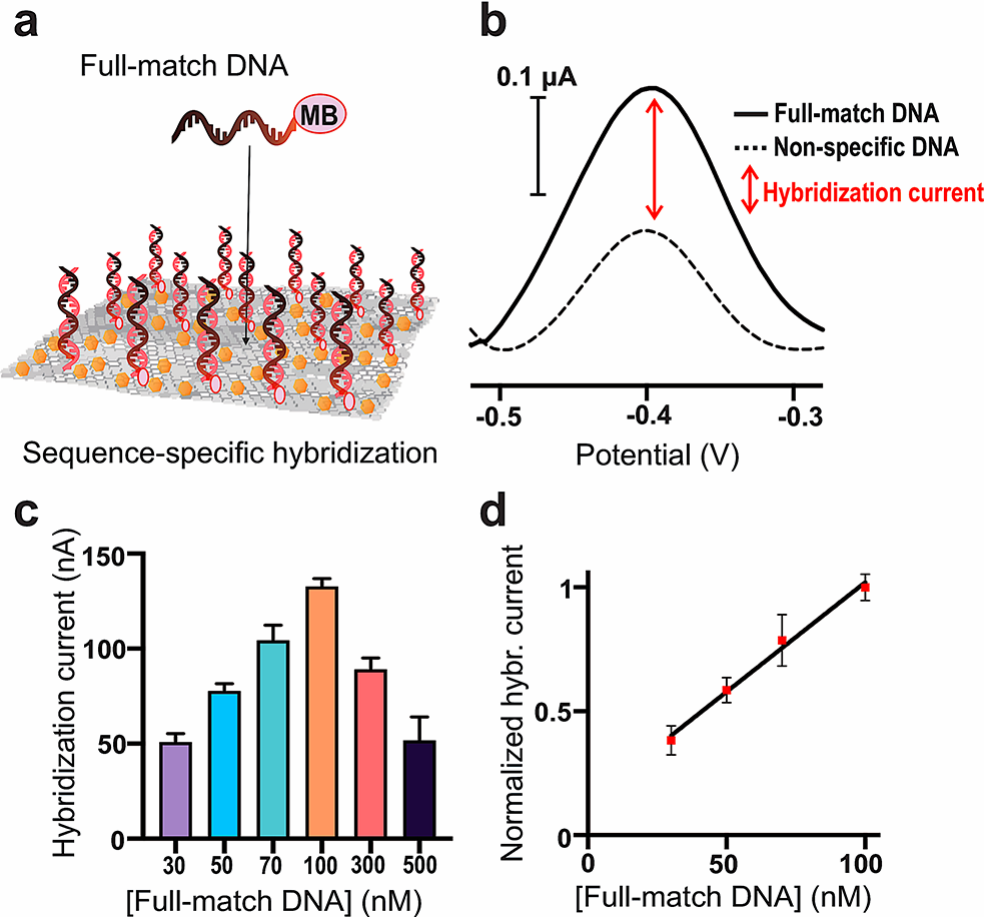
(a) Schematic drawing of the formation of specific DNA duplexes on the CNT electrode surface. MB is the Atto-MB2 redox tag. (b) Representative voltammograms obtained using a redox-tagged full-match DNA (100 nM) and a redox-tagged nonspecific DNA (100 nM), respectively. The difference between the two signals gives the hybridization current specific to the formed duplexes. (c) Hybridization current values obtained using different concentrations of full-match DNA (mean value ± sd, *n* = 3). (d) Linear fit of normalized hybridization currents for full-match DNA concentrations in the 30–100 nM range. Hybridization currents were normalized with respect to the most intense response recorded at the 100 nM concentration level.

We then investigated the controlled formation of DNA duplexes at the electrode surface and studied how to correctly interpret the signal resulting from the specific hybridization process. In this case, we used a sample solution of a DNA oligonucleotide with a sequence full complementary (full-match DNA) to that of the capture probe anchored to the electrode surface and conjugated with an Atto-MB2 redox tag at its 3′-terminus. As illustrated in Figure 3a, the formation of a duplex brings the tag redox of the full-match DNA near the electrode surface, promoting an efficient electron transfer process. We incubated DNA-functionalized SWCNT-SPE with different concentrations (30 nM to 500 nm) of complementary Atto-MB2-labeled DNA strands and recorded the resulting current signal (Figure 3b,c). In parallel, we systematically carried out the same experiments using a sample solution of a noncomplementary DNA oligonucleotide, which at each concentration allowed us to estimate the contribution to the final signal given by the nonspecific adsorption of the DNA strands onto the CNT surface. In this way, it was possible to determine the portion of the total amperometric output that is exclusively attributable to the formation of a duplex between complementary strands at the interface of the materials, which we have called hybridization current (Figure 3b,c). The hybridization current increased linearly when increasing the full-match DNA concentration from 30 to 100 nM, reached a maximum of 133 ± 11 nA at 100 nM and then dropped at higher concentrations of 300 and 500 nM (Figure 3c). Based on the calculated probe density of 4.3 × 10^11^ DNA probes/mm^2^, which results in ∼9 picomoles of DNA probes available on the whole electrode surface, using 50 μL of incubating solution generates a local probe concentration of around 180 nM. This explains why a maximum value of hybridization current is achieved when using a 100 nM full-match DNA solution. For the same reason, at higher concentrations of DNA, the CNT surface reaches a saturation point in terms of duplexes assembled at the interface, and a further contribution to the total current signal is given only by an increase in the nonspecific surface adsorption of the full-match DNA, leading to reduced values of the hybridization current (Figure 3c).

The hybridization current is a linear function of the concentration of full-match DNA in the 30–100 nM range, for which it was possible to determine the equation *i*_*n*_ = 0.14 (±0.05) + 0.0088 (±0.0007) [full-match DNA] (nM), where *i*_*n*_ is expressed as normalized hybridization current (Figure 3d). Based on the reported equation, a limit of detection (LOD) of 8 nM and a limit of quantification (LOQ) of 27 nM were calculated, respectively. These results show that an ideal working range for DNA-functionalized SWCNT-SPEs as hybridization platforms is identified in which the amperometric output can be expressed as the current signal derived exclusively from the formation of specific duplexes at the interface. Controlling hybridization processes at the interface enables the design of CNT-based electrochemical platforms in which programmable dynamic DNA systems can be engineered to provide an amperometric output in response to a nucleic acid input. DNA duplexes formed on the electrode surface can be designed to undergo strand displacement reactions in the presence of a third input strand. Toehold-mediated strand displacement is likely the most simple and versatile programmable process to carry out molecular operations enabling applications in sensing, information processing, and synthetic biology.^40−43^

### Toehold-Mediated Strand Displacement Reactions

**Figure 4:**
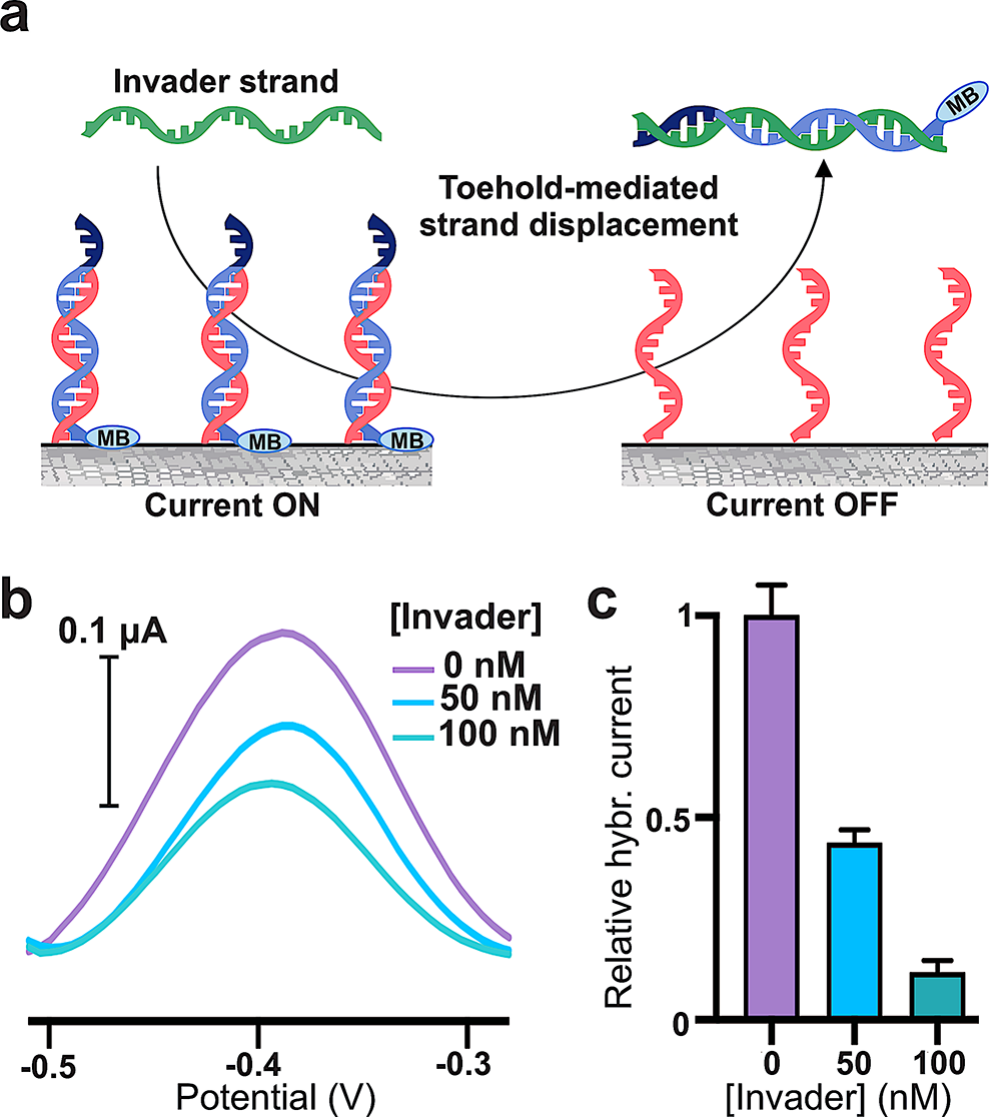
(a) Schematic illustration of a toehold-mediated DNA strand displacement reaction conducted on the surface of SWCNT-SPEs, which can be monitored via amperometric measurements as an invader strand removes a redox-tagged strand near the electrode surface. MB is the Atto-MB2 redox tag. (b) Representative voltammograms recorded in the presence of different concentrations of the invader strand. (c) Relative hybridization current values measured in the presence of different concentrations of the invader strand (mean value ± sd, *n* = 3).

In the present work we engineered a strand displacement amperometric platform by assembling DNA duplexes on the CNT surface in which one strand is tagged with Atto-MB2 and presents a 6-nt-long toehold domain (Figure 4a).

A full complementary input strand to this latter, represented as the invader strand in Figure 4a, can then trigger a toehold-mediated strand displacement reaction leading to removal of the redox tag from the vicinity of the electrode surface. This process can be monitored directly through an amperometric measurement. Starting from duplexes obtained using a 100 nM concentration of redox-tagged oligonucleotide, the incubation of the electrode surface with different concentrations of the invader strand caused a reduction of the total current signal in accordance with the strand displacement mechanism (Figure 4b).^44^ This translated in values of hybridization current, calculated as described above, which decreased proportionally with increasing concentrations of invader strand, with the strand displacement reaction proceeding at an almost quantitative rate (hybridization current reduced by 88% compared to that recorded in the absence of the invader strand) when using a 100 nM invader strand (Figure 4c). We then investigated the response of the strand displacement mechanism when in the presence of nontarget sequences. Three invader strand sequences were selected that were a completely random sequence, a three-mismatch sequence, and a single-mismatch sequence, these two latter bearing three or one noncomplementary nucleobases in the toehold region, respectively (sequences are reported in the SI). Incubation with the random sequence resulted in no decrease of the signal (Figure S5). In the case of the three-mismatch sequence, the reduction in the relative hybridization current was approximately 10%, whereas a more significant reduction in the relative hybridization current of around 70% was observed when in the presence of the single-mismatch sequence (Figure S5). Nevertheless, the hybridization current in this last case was still three times higher than that obtained, instead, when in the presence of a full-complementary invader strand at the same concentration, which shows that the presence of a single noncomplementary nucleobase in the invader strand leads to a decrease in the hybridization current to values that can still be distinguished from those given by a full-complementary invader sequence. It is also worth pointing out that the effect of mismatches in complementary DNA strands depends on the nature of the nucleobase substitution and on their position within the sequence.^45^ For this reason, each sequence should be investigated independently when assessing the effect of mismatches on duplex stability and strand displacement efficiency. Since the sequences of the DNA strands used to form the functional duplexes on the electrode surface can be rationally designed to allow for a strand displacement reaction with a desired invader oligonucleotide sequence, it should be noted that it is possible to engineer SWCNT-SPEs into preloaded amperometric platforms with DNA duplexes that respond via a signal-off mechanism specifically to the presence of a target nucleic acid.

### Artificial DNA Duplexes Templated by Melamine Enabling Toehold-Free Strand Exchange Reactions

**Figure 5:**
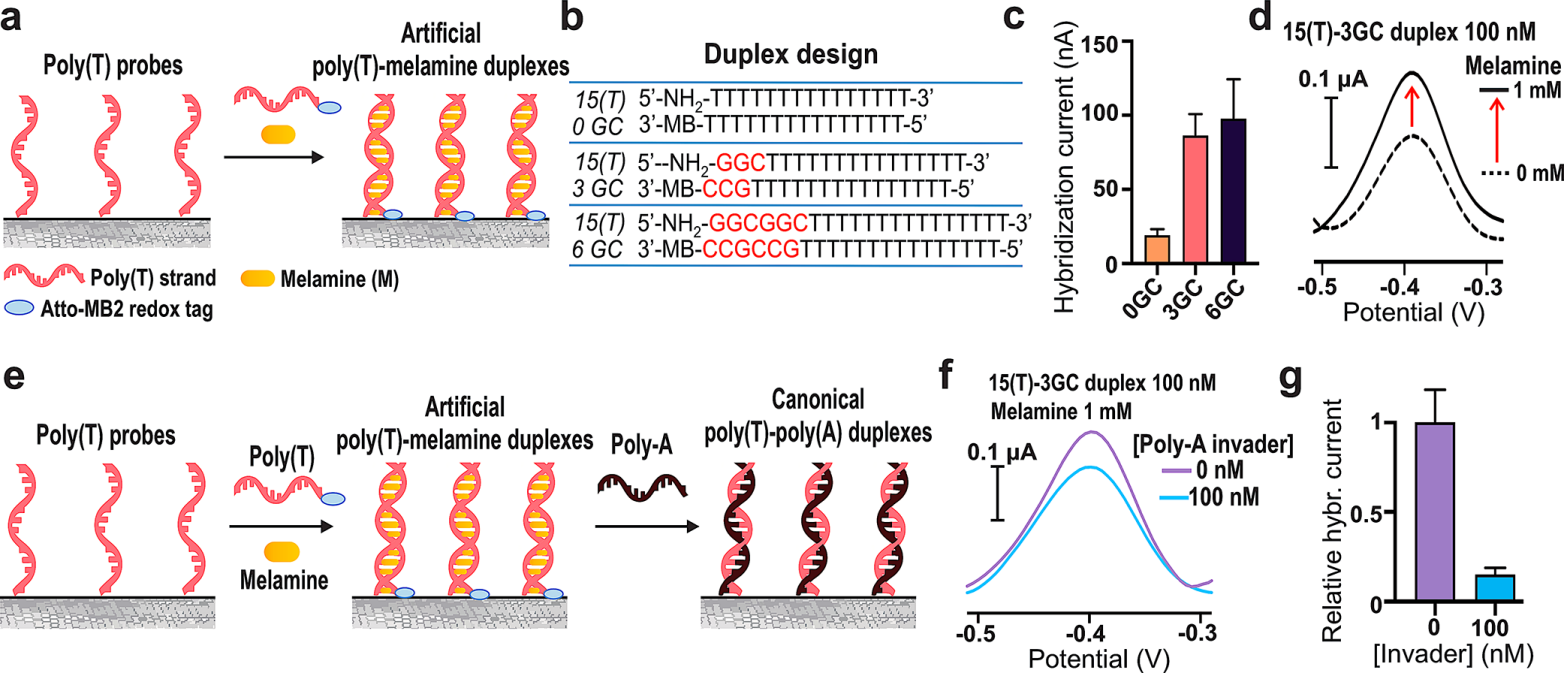
(a) Schematic drawing of the controlled formation of artificial poly(T)-melamine duplexes on the surface of SWCNT-SPEs. (b) Table reporting the DNA sequences designed for the controlled assembly of poly(T)-melamine duplexes on the electrode surface. (c) Hybridization current values related to the artificial poly(T)-melamine duplexes obtained using 100 nM of 15-nt poly(T) sequences (0GC), 15-nt poly(T) sequences conjugated with 3 G/C bases (3GC), 15-nt poly(T) sequences conjugated with 6 G/C bases (6GC), respectively, in the presence of 1 mM melamine (mean value ± sd, *n* = 3). (d) Representative voltammograms obtained using 15(T)-3GC-duplex-forming strands (100 nM) in the presence (solid line) and in the absence (dashed line) of melamine (1 mM), respectively. The difference between the two signals is the hybridization current specific to the formed artificial poly(T)-melamine duplexes. (e) Schematic illustration of a strand exchange reaction conducted at the electrode surface, where a poly(A) strand replaces the central melamine units of an initial poly(T)-melamine duplex to form a canonical poly(T)-poly(A) duplex. (f) Representative voltammograms recorded in the presence (blue line) and in the absence (violet line) of a poly(A) sequence reacting with poly(T)-melamine duplexes previously assembled on the electrode surface. (g) Relative hybridization current values obtained upon incubation with a poly(A) invader strand (100 nM), showing that the induced strand exchange reaction is almost quantitative (mean value ± sd, *n* = 3).

Based on our ability to control DNA hybridization processes on the CNT electrode surface, we therefore set out to explore the possibility to use more complex nucleic acid structures and design amperometric hybridization platforms that support nucleic acid operations complementary to conventional toehold-mediated strand displacement reactions. For this purpose, we focused on small molecule-templated artificial DNA duplexes that were recently fully characterized by Chengde Mao and collaborators.^46^ These are poly(thymine) (poly(T)) strands that self-associate into antiparallel, right-handed duplexes in the presence of a central pile of stacked melamine molecules as the latter provide a geometry of hydrogen bonding sites mimicking that of adenine. It was demonstrated that these structures are highly dynamic and can support toehold-free strand exchange reactions where poly(A) sequences, which bind tighter to poly(T) strands, replace the central melamine units causing a transition from the artificial melamine-templated duplex to a canonical poly(T)-poly(A) duplex.^46^ Our first goal was then to assemble artificial poly(T)-melamine duplexes on the surface of SWCNT-SPEs (Figure 5a). Since the controlled assembly of these types of structures on the surface of materials substrates using two distinct poly(T) strands had never been reported, we decided to combine the functionalization protocols developed in this work with the rational design of a series of nucleic acid sequences to test.

One strand was modified with an amine group and then anchored to the electrode surface, whereas the other was conjugated with Atto-MB2 to generate a current signal following the hybridization process (Figure 5b). In this regard, it should be noted that melamine is not electroactive in the same potential window of the Atto-MB2 redox tag and does not lead to any interference with the measured current signal (Figure S6). We have chosen to work with 15 bases long poly(T) strands because these can assemble into stable artificial duplexes in the presence of >5 equiv of melamine in solution and can form poly(T)-poly(A) duplexes as well at room temperature.^46^ In addition to 15-nt homopoly(T) strands, we designed 18-nt and 21-nt sequences in which the 15-nt poly(T) core was flanked by additional 3 or 6 G or C bases, respectively (Figure 5b). This would allow us to investigate whether the presence of a set of bases that give the canonical Watson–Crick hydrogen bonding could provide additional driving force for the formation of artificial poly(T)-melamine duplexes on the electrode surface. For each sequence pair, the CNT electrode surface was functionalized with the amine-modified strand (500 nM), treated with pyrene (500 nM in DMSO) as a backfilling agent and eventually incubated with a complementary poly(T)-based strand (100 nM) in the presence of melamine (1 mM). The amperometric output specific to the formation of artificial poly(T)-melamine duplexes at the interface, that is, the hybridization current, was calculated as the difference between the signal recorded in the presence and in the absence of melamine (Figure 5c,d). Indeed, in this case, the experiments performed in the absence of melamine lead to current signals that are given exclusively by the nonspecific adsorption of the redox-tagged DNA strands on the electrode surface, allowing to calculate the hybridization current values in the same way as described above. When mirror image 15-mer poly(T) strands were used, the resulting hybridization current was quite low (19 ± 7 nA). In this case, we assume that artificial poly(T)-melamine duplexes can form both in solution and on the electrode surface, based on the same exact sequences. It is likely that self-assembly in solution was largely favored, making the concomitant surface formation of poly(T)-melamine structures just a marginal process. Conversely, the use of poly(T) sequences flanked by additional G and C bases led to hybridization current values around 100 nA, demonstrating that the introduction of complementary Watson–Crick base pairing is a helpful strategy to provide a driving force for the formation of poly(T)-melamine structures on the electrode surface. We observed that there was no statistically significant difference (*p* > 0.05) between the hybridization current obtained for the 3 GC- and the 6 GC-based molecular designs (Figure 5c), indicating that a set of 3 GC base pairs is sufficient to promote an interaction between the DNA probes anchored to the electrode surface and the strands in solution and to support the subsequent melamine-templated hybridization of the poly(T) regions (Figure 5d). Based on these results, we explored the possibility of performing and monitoring through direct amperometric measurements strand exchange reactions alternative to conventional toehold-mediated strand displacement reaction. To this aim, we engineered our hybridization amperometric platform by assembling poly(T)-melamine duplexes on the CNT surface using 5′-NH2-GGC-(T)_15_-3′ as a capture probe and 5′-(T)_15_-GCC-MB-3′ as its complementary sequence in the presence of melamine. A poly(A) 15-mer (poly(A) invader) can then serve as an input strand to break the preformed artificial poly(T)-melamine structures through the formation of thermodynamically favored canonical poly(T)-poly(A) duplexes, causing displacement of the redox tagged-strands and a reduction in the measurable current signal (Figure 5e). Starting from poly(T)-melamine duplexes obtained using a 100 nM concentration of the redox-tagged sequence, the incubation of the electrode surface with an equimolar concentration of the poly(A) invader led to a decrease in the total current signal according to the proposed mechanism (Figure 5f). The hybridization current, calculated as mentioned above, recorded upon addition of the poly(A) invader, was approximately 15% of that measured in its absence (Figure 5g), which shows that the strand exchange reaction conducted at the electrode surface was highly efficient. Of note, this is the first time that dynamic reactions of this type based on artificial poly(T)-melamine duplexes have been performed on the surface of a materials substrate.

## Conclusion

This study provides design rules and a research protocol for interfacing dynamic DNA systems with CNT-based electrodes. We have demonstrated that a strategy to limit unwanted nonspecific interactions between DNA strands and the CNT surface is key to developing a DNA/electrode interface that supports the unambiguous electrochemical detection of nucleic acid recognition events. Our results point out that a systematic evaluation of the nonspecific signal due to DNA physisorption on the electrode surface is instrumental to a correct interpretation of the electrochemical output specifically generated by the hybridization processes involving a target sequence. The protocols developed in the present work demonstrate that DNA strand displacement reactions can be designed and conducted on the CNT electrode surface and can be monitored through measurements of the specific hybridization current. This suggests that it will be possible to interface CNT-SPEs with more complex dynamic systems enabled by DNA nanotechnology for the engineering of new formats of E-DNAs and amperometric biosensors.^7,47,48^ We applied our strategy to the assembly of artificial DNA structures based on poly(T) duplexes templated by the small molecule melamine. These allow for performing toehold-free strand exchange reactions triggered by a poly(A) sequence, which can be easily followed by measuring the resulting hybridization current. DNA structures based on poly(T) duplexes templated by the small molecule melamine are a recent discovery that has the potential to widen the available tools for programming DNA-based molecular systems and interfaces. The possibility to perform toehold-free strand exchange reactions that leverage a basic poly(A) sequence as the invader strand is particularly appealing for the development of sequence-independent electrochemical platforms enabling detection of non-nucleic-acid analytes. Many molecular systems have been recently developed in which generation or release of an arbitrary nucleic acid sequence can be controlled upstream by a specific protein, including antibodies, transcription factors, and other functional proteins.^41,49−53^ An electrochemical platform designed to respond to an arbitrary protein-controlled poly(A)-based input by simple disassembly of poly(T)-melamine structures would require no efforts in sequence design, and it could be applied to any molecular system engineered to provide a poly(A) strand as a molecular output. Furthermore, more complex architectures based on the same strategy could be designed in which toehold-mediated and toehold-free reactions are performed in an orthogonal way, facilitating the design of molecular interfaces for multiplex analyses. Our results suggest also that alternative structures based on a similar principle, that is, small molecule- or metal-templated nucleic acid structures, may be controlled at the electrode surface, which could be useful in the design of new formats of sensors and portable electrochemical devices.^54,55^ We envision that functional interfaces that use programmable DNA systems in conjunction with carbon nanotube substrates will support the development of new generations of electrochemical sensors and diagnostic devices integrating the advantageous physicochemical properties of nanostructured carbon-based materials with specific and predictable nucleic acid hybridization.

## Supporting Information Available

DNA sequences, enzyme-based electrochemical signal, calculation of probe density, efficacy of electrode backfilling using pyrene, strand displacement using mismatched sequences, and melamine electroactivity in the potential range of interest (PDF)
