# Supplementary material for: Controlling Dynamic DNA Reactions at the Surface of Single-Walled Carbon Nanotube Electrodes to Design Hybridization Platforms with a Specific Amperometric Readout
Source: Anal Chem. 2022 Mar 18;94(12):5075–83. doi: 10.1021/acs.analchem.1c05294 (PMC8968946; doi:10.1021/acs.analchem.1c05294)
Supplement: Supplementary file 1 — ac1c05294_si_001.pdf [file ac1c05294_si_001.pdf]

# SUPPORTING INFORMATION

## Controlling dynamic DNA reactions at the surface of single-walled carbon nanotube electrodes to design hybridization platforms with specific amperometric readout

Simone Fortunati,[a] Ilaria Vasini,[a] Marco Giannetto,[a] Monica Mattarozzi,[a] Alessandro Porchetta,[b] Alessandro Bertucci,*[a] and Maria Careri[a]

[a] Department of Chemistry, Life Sciences and Environmental Sustainability, University of Parma, 43124 Parma, Italy

Email: alessandro.bertucci@unipr.it

[b] Department of Chemical Sciences, University of Rome Tor Vergata, 00133 Rome, Italy

## Table of contents

1. DNA sequences

2. Supporting Figures

# 1. DNA sequences

**Capture Probe Biotin 15-mer**
5'-C6 Amino-TTTTTTTTTTTTTTT-Biotin-3'

**Capture Probe poly(T) 15-mer.**
5'-C6 Amino-TTTTTTTTTTTTTTT-3'

**Cy3-labelled DNA**
5'-C6 Amino-GCAGCTAAGCAGGCGGCTCACAAAACCATTCGCATGCGGC-Cy3-3’

**Redox-tagged poly(T) 15-mer**
5'-TTTTTTTTTTTTTTT-AttoMB2-3'

**Redox-tagged poly(A) 15-mer**
5'-AAAAAAAAAAAAAAA-AttoMB2-3'

**Redox-tagged poly(A)-toehold 21-mer**
5'-CAGACGAAAAAAAAAAAAAAA-AttoMB2-3'

**Full complementary invader 21-mer**
5'-TTTTTTTTTTTTTTTCGTCTG-3'

**Random sequence invader 21-mer**
5'-CGATTACACGTAGCTATCGAT-3'

**3-Mismatch invader 21-mer**
5'-TTTTTTTTTTTTTTTTATATG-3'

**1-Mismatch invader 21-mer**
5'-TTTTTTTTTTTTTTTCGTATG-3'

**Capture Probe poly(T)-3GC 18-mer**
5'-C6 Amino-GGCTTTTTTTTTTTTTTT-3'

**Capture Probe poly(T)-6GC 21-mer**
5'-C6 Amino-GGCGGCTTTTTTTTTTTTTTT-3'

**Redox-tagged probe poly(T)-3GC 18-mer**
5'-TTTTTTTTTTTTTTTGCC-AttoMB2-3'

**Redox-tagged probe poly(T)-6GC 21-mer**
5'-TTTTTTTTTTTTTTTGCCGCC-AttoMB2-3'

**Invader poly(A) 15-mer**
5'-AAAAAAAAAAAAAAA-3'

## 2. Supporting Figures

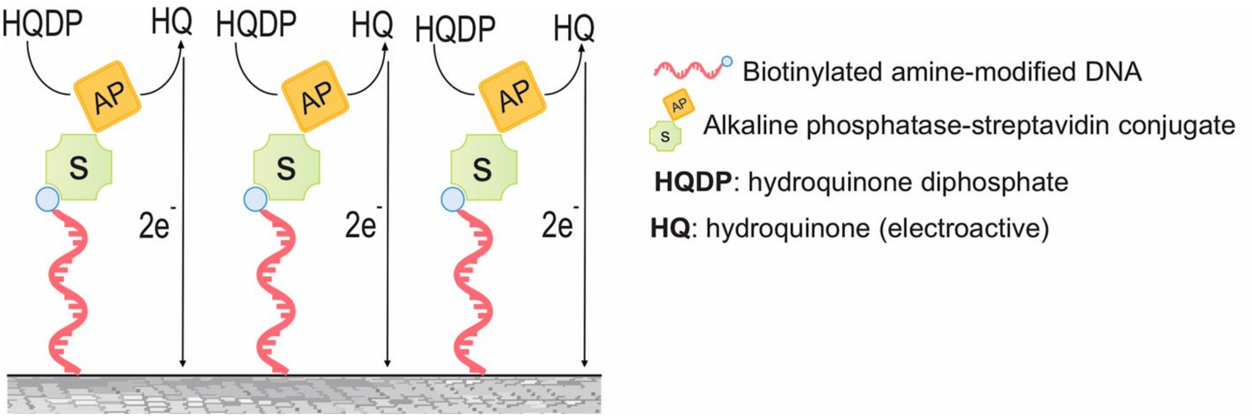


**Figure S1** Schematic drawing of the generation of an enzyme-based amplified current signal using biotinylated DNA probes complexed with alkaline phosphatase-streptavidin conjugates.

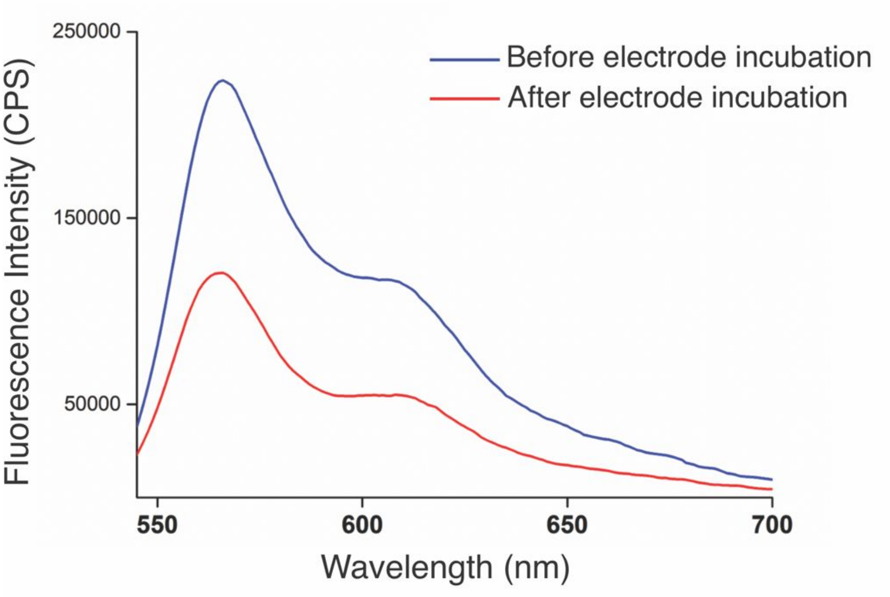


**Figure S2** Emission spectra of a Cy3-labelled amine-modified DNA solution in carbonate buffer (500 nM) before (blue line) and after (red line) incubation on an NHS-functionalized SWCNT-SPE ($\lambda_{ex}$ = 540 nm). The probe density was estimated as follows using the average fluorescence intensity at $\lambda_{em}$ = 565 nm from three independent replicates.

(1) $$\Delta_{CPS} = 205669 - 131594 = 74075 \Rightarrow \frac{74075}{205669} \times 100\% = 36\%$$

(2) $$moles\ \text{Cy3DNA} = (500 \times 10^{-9}\ M) \times (50 \times 10^{-6}\ L) = 2{,}5 \times 10^{-11}\ mol$$

(3) $$DNA\ moles\ attached\ to\ the\ surface = (2{,}5 \times 10^{-11}\ mol) \times 36\% = \\ = 9 \times 10^{-12}\ mol$$

(4) $$Electrode\ surface\ area = \pi(4\ mm)^2 = 12{,}6\ mm^2$$

(5) $$\frac{moles}{electrode\ surface} = \frac{(9 \times 10^{-12}\ mol)}{(12{,}6\ mm^2)} = 7{,}2 \times 10^{-13} \frac{mol}{mm^2}$$

(6) $$\frac{DNA\ molecules}{electrode\ surface} = \frac{(9 \times 10^{-12}\ mol) \times N_A}{(12{,}6\ mm^2)} = 4{,}3 \times 10^{11} \frac{molecules}{mm^2}$$

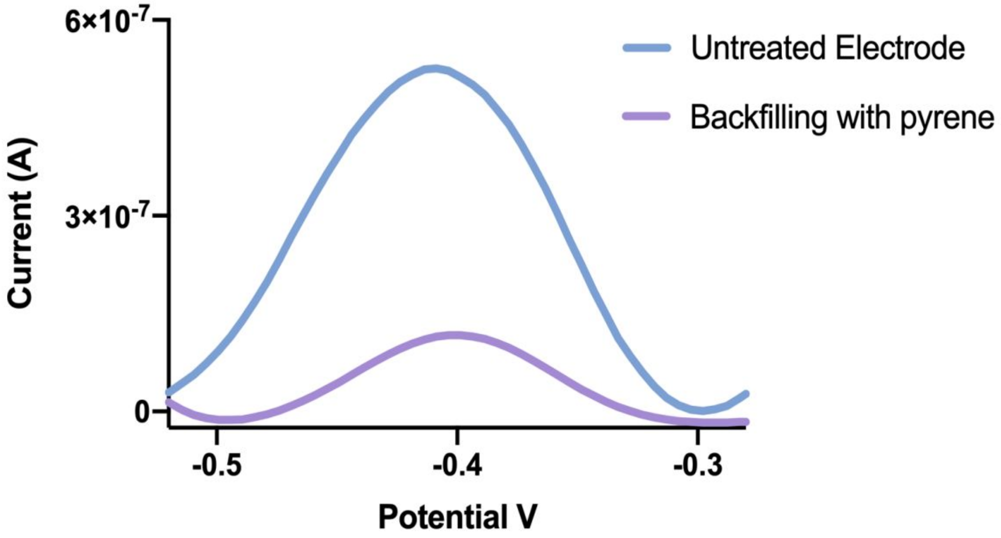


**Figure S3** Voltammograms recorded after incubation of 100 nM non-specific poly(T) DNA labelled with the Atto-MB2 redox tag for 2 h on an untreated electrode (blue curve) or an electrode treated with pyrene 500 nM (violet curve).

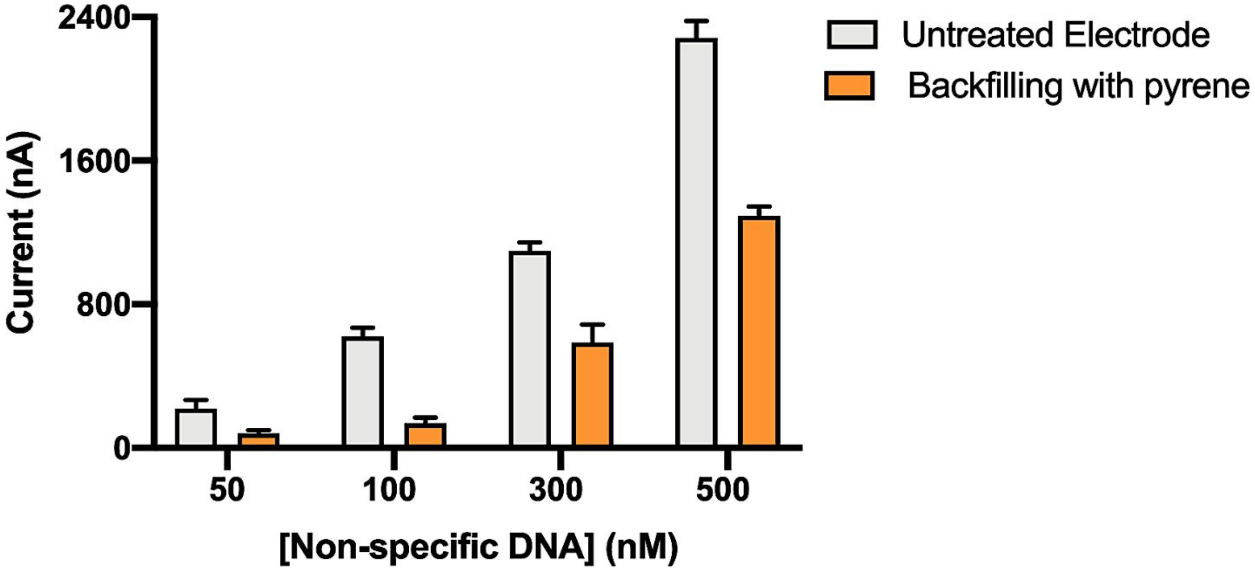


**Figure S4** Non-specific current signal attributable to the physisorption of DNA on the electrode surface using untreated electrodes (grey bars) and electrodes treated with pyrene as a backfilling agent (orange bars) at different concentrations of a redox-tagged poly(T) DNA sequence (mean value ± sd, n = 3).

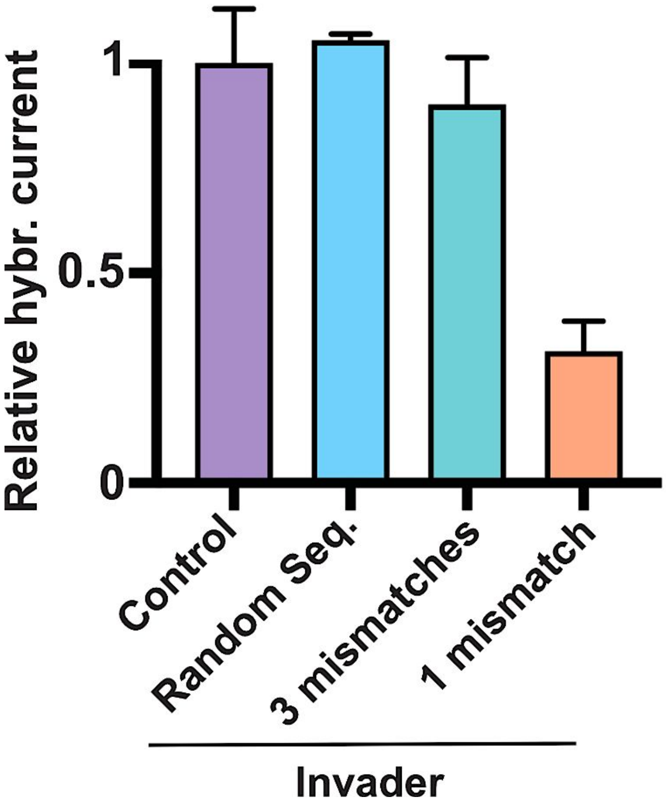


**Figure S5** Normalized hybridization currents obtained performing toehold-mediated strand displacement experiments using a single-mismatch invader strand (orange), a three-mismatch invader strand (green), or a random sequence invader strand (blue), all at a concentration of 100 nM, with respect to when in the absence of an invader probe (Control, violet) (mean value ± sd, n = 3).

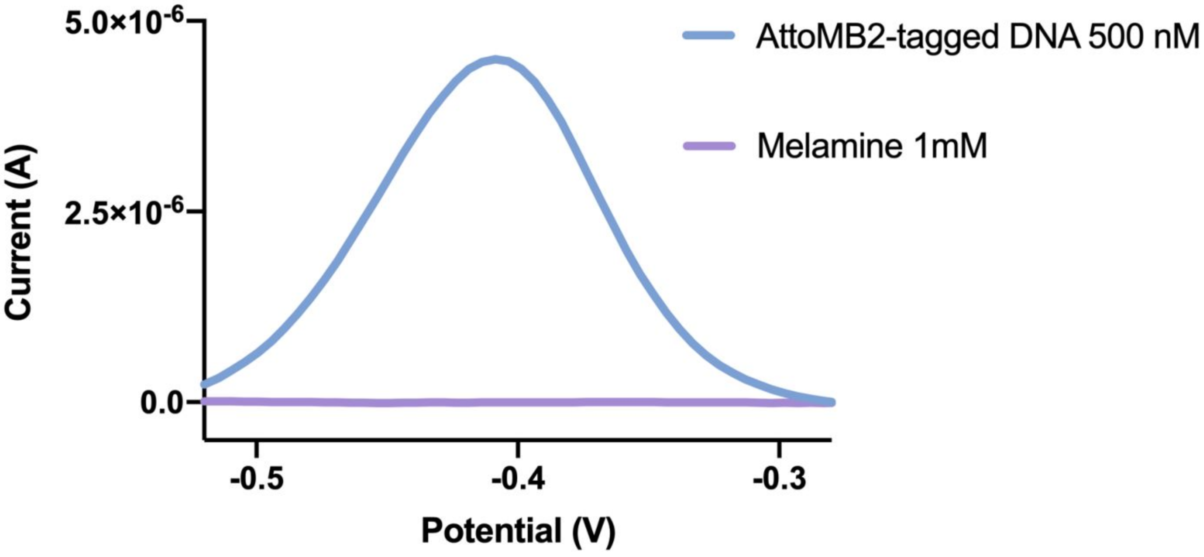


**Figure S6** Voltammograms of an aqueous solution of 500 nM electroactive AttoMB2-tagged DNA (blue curve) or of 1mM melamine (violet curve) incubated on a SWCNT-SPE. Melamine is not electroactive in the redox potential window of AttoMB2.
